# Attention Deficit Hyperactivity Disorder: A Risk Factor for Premature Discontinuation of Inpatient Opioid Withdrawal Treatment

**DOI:** 10.3390/jcm13113301

**Published:** 2024-06-03

**Authors:** Nikolas Gaspar, Laura Luisa Kilarski, Helena Rosen, Maximilian Huppertz, Alexandra Philipsen, Henrik Rohner

**Affiliations:** Department of Psychiatry and Psychotherapy, Faculty of Medicine, University of Bonn, 53111 Bonn, Germany

**Keywords:** ADHS, SUD, opioid, addiction, withdrawal treatment

## Abstract

**Background**: Substance use disorders present a tremendous challenge within contemporary healthcare systems. Specifically, in the domain of opioid use disorders (OUDs), several foundational elements are crucial for the efficacious management of afflicted individuals. Regrettably, the premature discontinuation of inpatient opioid withdrawal treatment is a prevalent phenomenon. This study aims to elucidate the prevalence of the premature termination of inpatient opioid withdrawal treatment among patients with comorbid ADHD. **Methods**: We conducted a comprehensive assessment of all participants currently undergoing inpatient opioid withdrawal treatment. Our assessment protocol included the administration of the ADHD Self-Report Scale (ADHD-SR) and the Wender Utah Rating Scale (WURS-k). Additionally, participants who met the thresholds on one or both questionnaires underwent further evaluation using the Diagnostic Interview for ADHD in Adults (DIVA-2.0). **Results**: The prevalence of individuals diagnosed with ADHD within the studied cohort was determined to be 29.3%. Among the subset of participants identified as ADHD-positive, a notable 54.5% prematurely ceased therapy. In contrast, among those identified as ADHD-negative, the premature discontinuation rate was substantially lower at 28.3%. **Conclusions**: In summary, the impact of ADHD as a comorbid condition on the efficacy of inpatient opioid withdrawal treatment has been underscored. By identifying comorbid ADHD early in the treatment process, tailored therapeutic approaches may help to maximize the effectiveness of interventions and may improve patient outcomes. This underscores the importance of proactive screening for ADHD as a psychiatric comorbidity in optimizing the management of individuals undergoing inpatient opioid withdrawal treatment.

## 1. Introduction

Substance use disorders (SUDs) pose significant challenges within contemporary healthcare systems, with opioid use disorders (OUDs) standing out as particularly pressing [1,2]. When discussing OUDs, they predominantly refer to a dependence on diacetylmorphine, commonly known as heroin [3]. Addressing the challenges of treating patients with OUD requires a multifaceted approach, prominently featuring outpatient opioid maintenance therapy for stabilizing individuals struggling with opioid dependency [4]. Historically, opioid maintenance therapy primarily utilized methadone [5]. However, subsequent research has revealed that levomethadone is the active enantiomer in methadone, prompting the widespread adoption of this substance for opioid maintenance therapy due to its reduced side effects [6]. Over time, additional pharmacological agents have been introduced, including buprenorphine, which has emerged as a frequently employed alternative owing to its diminished sedative properties compared to levomethadone [7]. Moreover, certain countries offer opioid maintenance therapy utilizing diacetylmorphine prescribed by physicians, permitting supervised intravenous self-administration by patients [8]. Despite the array of available substances, ongoing research endeavors continually introduce novel compounds, such as extended-release morphine tablets, aimed at maximizing individualized treatment modalities for affected individuals [9]. However, when opioid consumption persists despite such interventions, the role of inpatient withdrawal treatments becomes pivotal, either mitigating the adverse consequences of sustained use or facilitating complete cessation [10,11]. Yet, transitioning towards abstinence necessitates long-term residential treatment, emphasizing the complexity of managing OUDs [12].

Both physical and psychiatric comorbidities play crucial roles in shaping the trajectory of SUDs [13]. Psychiatric ailments often precipitate self-medication practices, potentially exacerbating primary condition symptoms or instigating additional psychiatric comorbidities, like substance-induced psychosis [14,15,16]. Furthermore, psychiatric disorders characterized by heightened impulsivity, such as borderline personality disorder (BPD) and attention deficit hyperactivity disorder (ADHD), can impede treatment adherence, hindering efforts to address SUDs effectively [17,18,19].

The worldwide ADHD prevalence is approximately 2.5% in adults and 3.4% in childhood, rendering it among the most pervasive neurodevelopmental disorders in the pediatric and adolescent populations [20,21]. It is typified by a persistent pattern of inattention and/or hyperactivity/impulsivity leading to functional impairment across various contexts, manifesting behavior that is comparatively more inappropriate or disruptive relative to peers of a similar age [22]. While ADHD symptoms typically manifest in childhood, a considerable proportion of afflicted individuals continue to experience its symptomatology into adolescence and adulthood [23]. As delineated in the Diagnostic and Statistical Manual of Mental Disorders 5 (DSM-5), three primary presentations of ADHD exist [24]. Within adult ADHD cohorts, the combined presentation prevails most prominently (70%), succeeded by the predominantly inattentive presentation (18.3%) and the pre-dominantly hyperactive–impulsive presentation (8.3%) [25]. In patients with OUD, an ADHD prevalence of 20.9% was reported in a recent meta-analysis [13]. There is a global consensus among experts regarding the use of stimulants as the primary therapeutic approach for ADHD, endorsed for both adults and pediatric populations [26]. This consensus is further reflected in the recommendations outlined in the National Institute for Health and Care Excellence (NICE) guidelines [27].

ADHD, in particular, has been associated with a more severe course of substance use, social and mental health impairment, and an increased likelihood of developing SUDs at a younger age [19,28,29]. Recognizing the importance of early diagnosis, experts advocate for initiating the diagnostic process for ADHD among SUD patients promptly when there are neither serious withdrawal symptoms nor serious intoxication [30]. Studies have reported a significant prevalence of ADHD among patients with alcohol and opioid use disorders, underscoring the need to consider therapeutic strategies for both disorders in treatment plans [31].

Notably, patients with comorbid OUD and ADHD undergoing oral opioid maintenance treatment exhibit many psychiatric comorbidities and greater addiction severity [32]. The adherence of individuals with concurrent ADHD and OUD undergoing Oral Opioid Maintenance Therapy is documented to be significantly suboptimal, frequently leading to discontinuations and the subsequent reinstatement of this therapeutic regimen [33,34,35]. Evidence indicates that pharmacological intervention for childhood ADHD utilizing stimulants may mitigate the risk of the subsequent development of SUDs [36]. However, there is an ongoing debate regarding the optimal management of patients with comorbid SUD and ADHD, due to concerns over the pronounced likelihood of abusing stimulants. This apprehension stems from the potential exacerbation of addictive propensities, particularly in individuals already susceptible to SUDs [30]. Nevertheless, psychopharmacological treatment alone appears insufficient in treating OUDs or ADHD in patients with ADHD actively using substances, highlighting the necessity of multimodal therapies [37]. Indeed, it is evident that no gold standard has been established for the treatment of patients presenting with an OUD alongside comorbid ADHD. Nonetheless, clinical recommendations advocate for the initiation of ADHD therapy promptly following the stabilization of OUD [38].

A critical question arises regarding the behavior of patients with comorbid ADHD during inpatient opioid withdrawal treatment. The premature discontinuation of treatment is a significant challenge during inpatient withdrawal treatments, often driven by unbearable withdrawal symptoms or strong cravings for opioids [39]. Given the impulsivity associated with ADHD, it is reasonable to hypothesize that patients with comorbid ADHD are more likely to prematurely terminate their inpatient withdrawal treatment [40].

Despite the importance of diagnosing ADHD among patients with SUD, data on whether comorbid ADHD influences the premature termination of inpatient opioid withdrawal treatment are lacking. Thus, the aim of this study is to determine the prevalence of premature therapy discontinuations in inpatient opioid withdrawal treatments among patients with comorbid ADHD.

Objectives: The primary objective of this study is to ascertain the prevalence of ADHD among patients undergoing opioid withdrawal treatment and to investigate the impact of an ADHD diagnosis on the trajectory of opioid withdrawal treatment. Specifically, this study aims to elucidate the disparity in premature discontinuation rates between patients with a confirmed diagnosis of ADHD and those without such a diagnosis.

## 2. Materials and Methods

### 2.1. Procedures and Study Design

We conducted a study involving patients with OUD undergoing inpatient opioid withdrawal treatment at the psychiatric department of the university hospital of Bonn. Patient recruitment commenced subsequent to obtaining ethical approval from the local Ethics Committee of the University Clinic of Bonn.

Upon admission for opioid withdrawal treatment, each patient received comprehensive information regarding the objectives and procedures of the study. The inclusion criteria comprised an age of 18 years or older and a confirmed diagnosis of OUD according to the International Statistical Classification of Diseases and Related Health Problems–10 (ICD-10) [41]. The exclusion criteria encompassed the presence of severe withdrawal symptoms persisting throughout the entire treatment duration, thus rendering a diagnosis of ADHD unfeasible. Additionally, conditions resulting in significant cognitive deficits, such as Korsakoff syndrome or acute psychosis, led to exclusion [16,42].

Upon obtaining written informed consent, participants completed the German versions of the self-rating behavior questionnaire for ADHD (ADHD-SR) and the Wender Utah Rating Scale (WURS-k) [43,44]. Subsequently, the Diagnostic Interview for ADHD in Adults (DIVA 2.0) was administered to those meeting the cutoff criteria in one or both questionaries [45]. The ADHD diagnosis adhered to the guidelines outlined by the National Institute for Health and Care Excellence (NICE) [27].

Following this, participants were categorized into two groups based on their ADHD status: ADHD-positive and ADHD-negative. These groups were then longitudinally monitored throughout the withdrawal treatment period to evaluate the impact of ADHD on treatment outcomes. Subsequent to enrollment, the participants underwent a treatment akin to other individuals undergoing opioid withdrawal therapy. The initial dosing aimed to establish a regimen wherein patients remained free of withdrawal symptoms, gradually tapering the dose thereafter until opioid cessation or reaching a threshold that prompted transition to opioid maintenance therapy. Nursing staff closely monitored patients throughout, conducting vital sign assessments at least four times daily to preempt potential complications. Daily ward physician rounds, excluding weekends, and weekly senior physician assessments were standard. Following ADHD diagnosis during the study, deliberation occurred during the ward physician rounds, with a collaborative assessment performed by senior physicians to validate the clinical ADHD diagnosis. In addition, discussions were held regarding participants who exhibited elevated scores on the ADHD-SB and/or WURS-K measures but received a negative diagnosis for ADHD based on the DIVA-2.0 interview. This inquiry aimed to determine the veracity of these apparent false-negative outcomes or potential inaccuracies in the diagnostic determination provided by the DIVA-2.0 interview.

### 2.2. Psychometric Inventories

The study utilized the WURS-k as a retrospective questionnaire to assess ADHD symptoms between the ages of 8 to 12, and used the ADHD-SR to evaluate ADHD symptoms in adulthood. The cut-off scores for ADHD-SR and WURS-k were set at 18 out of 66 points and 30 out of 84 points, respectively, yielding a combined sensitivity of 94% and specificity of 56% [46].

For diagnostic purposes, DIVA 2.0 was employed. This clinical interview adhered to the DSM-IV criteria and encompassed inquiries regarding both childhood and adult symptoms of ADHD. DIVA 2.0 comprised nine questions each for inattentive symptoms and hyperactivity/impulsivity, with concrete examples provided to facilitate symptom identification. Additionally, the interview assessed the impact of ADHD symptoms on various domains of functioning. The diagnosis of ADHD required the presence of at least six symptoms in one cluster, with symptom onset before the age of seven [45]. DIVA 2.0 showed sufficient validity among SUD patients with comorbid ADHD [47]. The DIVA 2.0 interviews, typically lasting 30 to 90 min, were conducted by a single interviewer (N.G.).

Recruitment and diagnosis occurred between June 2022 and August 2023. The DIVA 2.0 interviews were scheduled subsequently. Statistical analysis was performed using IBM SPSS Version 29.0.2.0 (20).

## 3. Results

### 3.1. Clinical and Sociodemographic Characteristics of the Participants

The study included 75 participants in total, of which 68% were male with a mean age of 45.52 ± 8.71 years. In total, 86.7% of the participants were treated with outpatient opioid maintenance therapy before applying to the inpatient opioid withdrawal treatment. Among them, 56% were treated with levomethadone, 8% with buprenorphine, 6.7% with methadone, and 16% with intravenous diacetylmorphine. According to the DIVA 2.0 interviews, 29.3% fulfilled the diagnostic criteria for childhood ADHD and 25.3% for persisting adult ADHD. In 96%, ADHD had not been diagnosed before participating in this study. In total, 36% prematurely ceased inpatient opioid withdrawal treatment.

When looking at the group of participants who prematurely terminated the inpatient opioid withdrawal treatment, 74.1% were male and the mean age was 42.48 ± 10.08 years. In total, 81.5% of these participants were treated with outpatient opioid maintenance therapy before applying to the inpatient opioid withdrawal treatment. Among them, 48.1% were treated with levomethadone, 14.8% with buprenorphine, 7.4% with methadone, and 11.1% with intravenous diacetylmorphine. According to the DIVA 2.0 interviews, 44.4% fulfilled the diagnostic criteria for childhood ADHD and 37% for persisting adult ADHD.

Looking at the two groups based on their ADHD status:

ADHD-positive: 72.7% were male with a mean age of 42.5 ± 8.9 years. In total, 90.9% of these participants were treated with outpatient opioid maintenance therapy before ap-plying to the inpatient opioid withdrawal treatment. Among them, 54.5% were treated with levomethadone, 22.7% with buprenorphine, 9.1% with methadone, and 4.5% with intravenous diacetylmorphine. According to the DIVA 2.0 interviews, 86.4% fulfilled the diagnostic criteria for persisting adult ADHD. In total, 54.5% prematurely ceased inpatient opioid withdrawal treatment.

ADHD-negative: 34% were female and the mean age was 46.77 ± 8.4 years. In total, 84.9% of the participants were treated with outpatient opioid maintenance therapy before applying to the inpatient opioid withdrawal treatment. Among them, 56.6% were treated with levomethadone, 1.9% with buprenorphine, 5.7% with methadone, and 20.8% with intravenous diacetylmorphine. In total, 28.3% prematurely ceased inpatient opioid withdrawal treatment. It should be noted here that four participants who met the cut-off criteria of the questionnaires discontinued treatment prior to the DIVA 2.0 interview. For diagnostic certainty, we classified these participants as ADHD-negative.

In summary, it is evident that the attrition rate is elevated among individuals with ADHD. Specifically, over fifty percent (54.5%) of patients presenting with comorbid ADHD prematurely terminate inpatient opioid withdrawal treatment, contrasting with a 28.3% discontinuation rate among ADHD-negative counterparts. Additionally, a noteworthy trend emerges wherein buprenorphine emerges as a relatively favored substitution in opioid maintenance therapy within the ADHD-positive cohort, whereas intravenous diamorphine employment remains comparably infrequent.

### 3.2. ADHD-SR, WURS-k, and DIVA 2.0

Overall, 38.7% met the cut-off criteria for ADHD in WURS-k and 54.7% met it in ADHD-SR, while 34.66% met the cut-off criteria for ADHD in both. Of the 40 DIVA 2.0 interviews, 55% confirmed the presence of an ADHD diagnosis, with the combined presentation being the most prevalent.

Looking at the group of participants who prematurely terminated the inpatient opioid withdrawal treatment, 48.1% met the cut-off criteria for ADHD in WURS-k and 63% met it in ADHD-SR, while 44.4% met the cut-off criteria for ADHD in both. Of the 14 DIVA 2.0 interviews, 85.71% confirmed the presence of an ADHD diagnosis, with the combined presentation being the most prevalent.

Looking at the two groups based on their ADHD status:

ADHD-positive: 77.3% met the cut-off criteria in WURS-k, 90.9% met it in ADHD-SR, and 68.18% met the cut-off criteria in both.

ADHD-negative: 22.6% met the cut-off criteria in WURS-k, 39.6% met it in ADHD-SR, and 20.75% met the cut-off criteria of both inventories.

The WURS-k and ADHD-SR appeared to be very useful diagnostic tools in this study, with a 45% false positive result rate and simple and low-threshold usability [44]. According to the standards of the international and German guidelines, ADHD diagnosis must be confirmed as a clinical diagnosis [30,48]. A structured diagnostic interview, like the DIVA 2.0 Interview, can aid diagnostic assessment and has been very useful in our research setting [45,47]. The combined presentation was the most prevalent (68.42%), followed by the predominantly hyperactive–impulsive presentation (15.78%) and the predominantly inattentive presentation (15.78%), in our OUD populations with comorbid ADHD in adulthood.

## 4. Discussion

The findings of this study underscore the significant impact of comorbid ADHD on the premature discontinuation of inpatient opioid withdrawal treatment. Our results reveal a striking prevalence of ADHD within the cohort of inpatients with OUD undergoing treatment, with 29.3% of participants diagnosed with ADHD. Notably, among those identified as ADHD-positive, over half (54.5%) prematurely ceased therapy, compared to a substantially lower premature discontinuation rate of 28.3% among ADHD-negative individuals. These findings highlight the pronounced vulnerability of individuals with comorbid ADHD to the premature termination of inpatient opioid withdrawal treatment.

Furthermore, it prompts inquiry into whether patients with ADHD exhibit a propensity to terminate treatment due to inherent symptomatology, such as impulsivity, or if they indeed encounter greater difficulty in coping with the withdrawal symptoms inherent to treatment. Additionally, an underlying factor may involve patients with ADHD facing inherent disadvantages during opioid withdrawal treatments, as the therapeutic team may encounter challenges in accommodating the unique needs of this patient population within the treatment milieu.

Our findings emphasize the critical need for systematic screening for ADHD among individuals undergoing opioid withdrawal therapy. The early identification of comorbid ADHD holds the potential to guide tailored therapeutic approaches, enhancing treatment efficacy and improving patient outcomes [30]. By addressing ADHD alongside opioid use disorder, healthcare providers can mitigate the risk of premature treatment discontinuation and optimize the effectiveness of interventions.

Moreover, our results underscore the importance of considering psychiatric comorbidities in addiction treatment settings [13]. The high prevalence of ADHD among individuals with opioid use disorder emphasizes the need for integrated treatment strategies that address both substance use and underlying psychiatric conditions [37]. A failure to address comorbid ADHD may compromise treatment outcomes and exacerbate the challenges associated with managing opioid-related disorders.

Our study contributes to the existing literature by elucidating the relationship between comorbid ADHD and treatment outcomes in the context of opioid withdrawal therapy. While previous research has highlighted the impact of psychiatric comorbidities on addiction treatment, our findings provide specific insights into the role of ADHD in premature treatment discontinuation. By addressing this gap in the literature, this study enhances our understanding of the complex interplay between psychiatric disorders and substance use disorders, informing future research and clinical practice.

Building upon the findings of this study, future research should explore the potential mechanisms underlying the association between comorbid ADHD and premature treatment discontinuation. Longitudinal studies are needed to examine the trajectory of ADHD symptoms during opioid withdrawal treatment and their impact on treatment outcomes over time. Additionally, investigations into the effectiveness of tailored therapeutic approaches for individuals with comorbid ADHD and opioid use disorder are warranted. By elucidating these mechanisms and interventions, future research can inform the development of targeted interventions to improve treatment outcomes for this vulnerable population.

Ultimately, the question as to whether patients with ADHD exhibit a pervasive trend towards low compliance arises, as this predisposes them to premature treatment cessation, non-adherence, or suboptimal therapeutic outcomes [49]. In psychiatric treatment paradigms, robust adherence assumes paramount importance, given the rapid deterioration of conditions resulting from medication noncompliance. This is particularly evident in schizophrenia, where noncompliance is frequently encountered, prompting an examination of whether individuals with comorbid schizophrenia and ADHD also demonstrate proclivity towards premature treatment discontinuation [50]. Similarly, affective disorders necessitate meticulous medication adherence to forestall disease exacerbation. The compliance behaviors of patients presenting with ADHD and comorbid affective disorders warrant investigation [51]. Moreover, within somatic disease contexts, adherence to treatment regimens assumes heightened significance, given the potential dire consequences of noncompliance, such as missed medication doses. Hence, the adherence patterns of individuals contending with comorbid ADHD and HIV infection, or those facing life-threatening cancer diagnoses alongside ADHD, merit scrutiny to ascertain these individuals’ ability to adhere to therapeutic protocols effectively [52].

Limitations: It is important to acknowledge the several limitations of our study. Firstly, the sample size may limit the generalizability of our findings to broader populations of individuals undergoing opioid withdrawal treatment. It is pertinent to acknowledge that the limited participant pool was influenced by the arduous nature of participant recruitment. Identifying suitable candidates for the study proved to be a formidable task, largely due to the intrinsic characteristics of opioid withdrawal treatment. Patients often presented in a highly intoxicated state upon admission, with subsequent occurrences of pronounced withdrawal symptoms during the course of treatment. These circumstances posed substantial challenges to enrollment, as the diagnostic evaluation of ADHD in such contexts was deemed impractical. Furthermore, prospective participants frequently exhibited a lack of interest in study involvement, focusing primarily on opioid withdrawal treatment or demonstrating a general apathy towards research participation. Additionally, our study relied on self-report measures for assessing ADHD symptoms, which may introduce bias or in-accuracies. In the intricate interplay of ADHD and SUD co-occurrence, individuals and clinicians alike encounter considerable difficulty in disentangling symptom attribution to either disorder. Within the clinical milieu of ADHD assessment, external collateral information assumes critical significance. However, within the SUD population, obtaining such data poses a formidable challenge. This predicament arises from disrupted familial connections and limited social networks, where associations primarily revolve around peers also grappling with SUD. Furthermore, the retrospective nature of the WURS-k may pose challenges in accurately capturing childhood ADHD symptoms. The attribution of childhood memories already poses a challenge for individuals with ADHD. For those enduring SUD over an extended period, this difficulty is compounded by the frequent phenomenon of memory retrieval failure. Future research should aim to address these limitations through larger sample sizes, objective measures of ADHD symptoms, and longitudinal study designs.

## 5. Conclusions

In conclusion, our study highlights the significant impact of comorbid ADHD on the premature discontinuation of inpatient opioid withdrawal treatment. By identifying ADHD as a risk factor for treatment non-adherence, our findings underscore the importance of systematic screening and integrated treatment approaches in addiction care settings. Moving forward, addressing comorbid psychiatric conditions such as ADHD alongside substance use disorders is essential for optimizing treatment efficacy and improving patient outcomes in addiction treatment settings. Future research endeavors should delve into elucidating the underlying factors contributing to treatment discontinuation in patients with and without ADHD undergoing opioid withdrawal therapy. Moreover, there is a pressing need to design studies aimed at evaluating interventions geared towards preventing the premature cessation of opioid withdrawal treatment. Furthermore, it is imperative that forthcoming investigations probe whether ADHD serves as a potential risk factor for treatment discontinuation across diverse therapeutic modalities. The potential avenues for inquiry may encompass critical interventions such as antiretroviral therapy for individuals afflicted with HIV infection or multimodal therapeutic approaches for cancer patients.

## Data Availability

The data that support the findings of this study are available from the corresponding author upon reasonable request.
